# Thermal and Circulatory Changes in Diverse Body Regions in Dogs and Cats Evaluated by Infrared Thermography

**DOI:** 10.3390/ani12060789

**Published:** 2022-03-20

**Authors:** Alejandro Casas-Alvarado, Julio Martínez-Burnes, Patricia Mora-Medina, Ismael Hernández-Avalos, Adriana Domínguez-Oliva, Karina Lezama-García, Jocelyn Gómez-Prado, Daniel Mota-Rojas

**Affiliations:** 1 Ph.D. Program in Biological and Health Sciences, [Doctorado en Ciencias Biológicas y de la Salud], Universidad Autónoma Metropolitana, Mexico City 04960, Mexico; ale0164g@hotmail.com; 2 Animal Health Group, Facultad de Medicina Veterinaria y Zootecnia, Universidad Autónoma de Tamaulipas, Victoria City 87000, Mexico; jmburnes@docentes.uat.edu.mx; 3 Facultad de Estudios Superiores Cuautitlán, Universidad Nacional Autónoma de Mexico (UNAM), Mexico City 54714, Mexico; mormed2001@yahoo.com.mx (P.M.-M.); mvziha@hotmail.com (I.H.-A.); 4 Neurophysiology, Behavior and Animal Welfare Assessment, DPAA, Universidad Autónoma Metropolitana (UAM), Xochimilco Campus, Mexico City 04960, Mexico; mvz.freena@gmail.com (A.D.-O.); kikislezama@hotmail.com (K.L.-G.); jocelyn.gomez.ilp@gmail.com (J.G.-P.)

**Keywords:** dogs, cats, thermal window, infrared thermography

## Abstract

**Simple Summary:**

Infrared thermography is a tool that measures changes in the surface temperature of the skin and has been used in companion animals to determine their health state and to diagnose inflammatory processes, neoplasia, pain, or neuropathies. Body regions such as the face, body, or hind/forelimbs are commonly used for thermography in dogs and cats. However, since there is disagreement about the differences in temperature recorded using this tool, this article analyzes the usefulness of IRT in companion animals (pets) as a complementary diagnostic method for evaluating thermal and circulatory changes. It analyzes the recent scientific evidence on the use of facial, body, and appendicular thermal windows in dogs and cats under different clinical conditions.

**Abstract:**

Infrared thermography (IRT) has been proposed as a method for clinical research to detect local inflammatory processes, wounds, neoplasms, pain, and neuropathies. However, evidence of the effectiveness of the thermal windows used in dogs and cats is discrepant. This review aims to analyze and discuss the usefulness of IRT in diverse body regions in household animals (pets) related to recent scientific evidence on the use of the facial, body, and appendicular thermal windows. IRT is a diagnostic method that evaluates thermal and circulatory changes under different clinical conditions. For the face, structures such as the lacrimal caruncle, ocular area, and pinna are sensitive to assessments of stress degrees, but only the ocular window has been validated in felines. The usefulness of body and appendicular thermal windows has not been conclusively demonstrated because evidence indicates that biological and environmental factors may strongly influence thermal responses in those body regions. The above has led to proposals to evaluate specific muscles that receive high circulation, such as the *biceps* *femoris* and *gracilis*. The neck area, perivulvar, and perianal regions may also prove to be useful thermal windows, but their degree of statistical reliability must be established. In conclusion, IRT is a non-invasive technique that can be used to diagnose inflammatory and neoplastic conditions early. However, additional research is required to establish the sensitivity and specificity of these thermal windows and validate their clinical use in dogs and cats.

## 1. Introduction

With the advent of new technologies to complement existing diagnostic methods, infrared thermography (IRT) has acquired greater importance in medicine and industry as a non-invasive method that does not use radiation [1,2]. IRT permits evaluating surface microcirculation based on consequent variations in tissue temperatures that medical research has associated with changes in the autonomic nervous system (ANS) of inflammatory, infectious, neoplastic, pain-induced, or stress-related origin [2,3,4]. Discussion of the medical use of IRT has centered on various anatomical regions that could be used as thermal windows to determine changes in surface blood flow that reflect an organism’s autonomic activity. The term thermal window refers to regions with surface blood vessels where changes can be detected that represent surface circulation [2]. Regions identified to date include the lacrimal caruncle, eye, ear, thorax flanks, femoral area, and face [5,6,7].

Studies of structures such as the lacrimal caruncle and eye have established that IRT strongly correlates with the activity of the ANS [8,9,10]. When the sympathetic element of the ANS is stimulated, an initial vasoconstriction response is produced due to the neurosecretion of adrenaline and noradrenaline, causing an alteration in the surface thermal response [2,7]. These findings demonstrate IRT’s usefulness for the early detection of inflammatory conditions [11], though its sensitivity is still in doubt because of potential biological and environmental influences [12]. This has been evidenced in appendicular regions, where sensitivity and effectiveness can be affected, for example, by the length or type of an animal’s hair or fur [13]. 

In the face of discrepant evidence on the effectiveness of the thermal windows currently used with dogs and cats, the aim of this review is to discuss the usefulness of IRT in household animals (pets) as a diagnostic method for evaluating thermal and circulatory changes under different clinical conditions, and then to analyze recent scientific evidence on the use of the facial, body, and appendicular thermal windows in dogs and cats.

## 2. Clinical Usefulness of IRT with Dogs and Cats

The IRT technique detects the radiation spectrum from an object between 7.5 and 13 μm using a specialized camera that produces a thermal image visualized and interpreted using special software [14]. IRT makes it possible to visualize surface changes in skin temperature that can be correlated with the body region evaluated [1], for example, in cases of osteoarthritis [15], spinal injury [16], conduct pass evaluations [11,17], infectious diseases [18,19], and malignant neoplasias [20,21].

Contrasting from existing clinical methodologies, IRT has the advantage of its non-invasiveness to assess the thermal state of animals without the need for physical or chemical restraint [2]. It is considered a non-contact complementary technique that does not require setting devices on the animals’ bodies [22] and may represent a useful method to avoid stress-induced hyperthermia due to handling [23,24]. Additionally, it is safe for animals [25], and the surface temperature changes can be evaluated in real-time with automated or handheld devices [26,27].

Hildebrandt et al. [28] observed that some types of lesions in local tissues are related to variations in blood flow that affect surface temperature, just as inflammation fosters hyperthermic states. IRT’s capacity to detect temperature variations is useful for evaluating skin wounds or lesions and detecting changes associated with hypothermia—degeneration, reduced muscular activity, decreased blood perfusion—among other areas of study where findings suggest that it can be used as a diagnostic instrument. This is the case, as well, of some neurological disorders [29,30], surgeries [2], degrees of pain [31], urological problems [32], cancer [20], and infectious diseases [19].

IRT is not limited to identifying areas where local temperature increases, as in most inflammatory processes such as dermal lesions and wounds. It can also recognize changes associated with decreased temperature due to tissue degeneration or reduced muscle activity. In patients with neurological disorders, the decline in surface temperature in the site of injury [29,30], as well as the detection of hypothermia associated with a blood perfusion failure in individuals with thromboembolic cases, can be determined by IRT [33]. Similarly, the drop in temperature during tissue surgery can aid in identifying the viable conditions of a graft, hypoperfusion states [2], or estimate the presence and degree of pain [31,34].

Thermal windows are used to evaluate these states, where changes in blood perfusion allow the thermal exchange with the environment [3,35,36,37]. However, in companion animals, the most appropriate body regions to quantify the superficial thermal response have not yet been established [38] because the sensitivity and specificity of these regions can be affected by internal and external factors that alter the vascular modulation of the ANS towards different stimuli [14]. Due to discrepancies in the available literature regarding thermographic research on different anatomical areas, it is necessary to establish the sensitivity and specificity of these regions to determine whether they represent a tool to recognize surface microcirculation mediated by the ANS activity [14]. Therefore, the current literature regarding the use and possible validation of each region applied in companion animals will be discussed below. 

## 3. Facial Thermal Windows

Studies of the facial region have described several thermal windows: the lacrimal caruncle [9,12,39,40], the eyeball [41], and the ear [22,42]. Some authors even suggest evaluating the entire area of the face [43]. In the cranial region (*regio cranii*), the auricula (*regio auricularis*) has been described as an area whose vascular response is associated with inflammatory, painful, and stressful events [22,42]. This region and the ocular window (*oculi*) in veterinary medicine are currently used as thermal windows to evaluate the infrared response [31,35,38]. 

**Figure 1:**
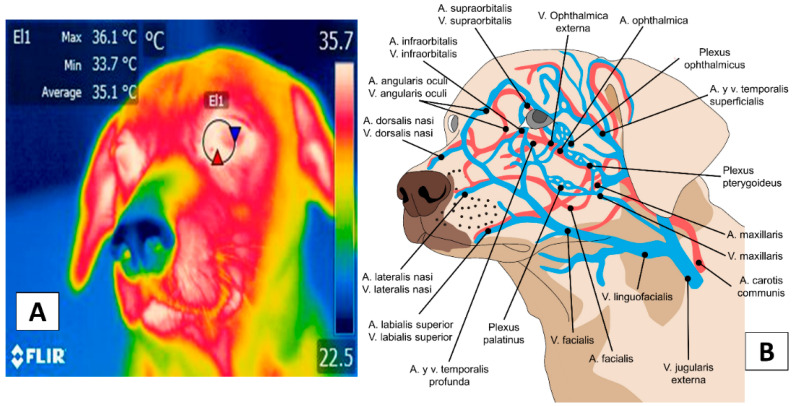
The thermal window of the lacrimal caruncle (El1). This window considers the region of the medial canthus of the upper and lower eyelids (**A**), which receive circulation from the supraorbital and infraorbital arteries (**B**). The latter holds sympathetic innervation from the infraorbital branch of the facial nerve, responsible for the autonomic hemodynamic activity of this region. Thermal images obtained using a FLIR thermal camera (Wilsonville, Oregon, U.S.).

The thermal window most widely analyzed in dogs is the caruncle (Figure 1) [44]. This region receives blood circulation from two main arteries, the supraorbital artery (*supraorbitalis*) and the infraorbital artery (*infraorbitalis*), from which emerges a capillary called the lacrimal (*lacrimalis*) that provides circulation to the lacrimal gland [45]. Its innervation is provided by the infraorbital nerve (*infraorbitalis*), derived as a branch of the facial nerve (*facialis*), considered a sensitive area that responds to the activity of the Sympathetic Nervous System (SNS) [41,46]. For this reason, the lacrimal caruncle has been extensively studied in animals to assess the ANS activity related to painful and stressful events [8,10,40,47,48].

**Figure 2:**
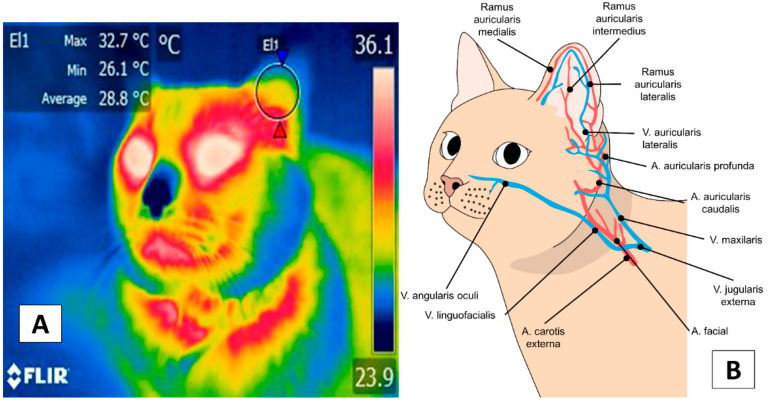
The thermal window of the ear. This window considers the auricular region marking a circle of approximately 2 cm (El1) to obtain the irradiated temperature of the tympanic membrane or the inner ear (**A**). This region presents irrigation from the deep auricular artery and three branches that allow thermal exchange with the environment (**B**) through vasomotor changes. Thermal images obtained using a FLIR thermal camera.

On the other hand, the pinna or ear region presents a vasculature coming from the caudal auricular artery (*auricularis caudalis*), dividing into three branches: the lateral (*lateralis*), intermediate (*intermedius*), and medial (*medialis*). The branch of the facial nerve (*facialis*), known as auriculopalpebral (*auriculopalpebralis*), innervates this region [45], as shown in Figure 2 [49].

Travain et al. [8], for example, evaluated the usefulness of IRT for measuring degrees of stress in 14 dogs, reporting that the surface temperature of the lacrimal caruncle increased during the examination period, in contrast to the measurements obtained before and after the clinical check-up. They determined that a reduction in the level of activity had occurred, so this response was more sensitive for determining stress of psychogenic type. The biological explanation is that, during stress events, sympathetic activation causes an increase in temperature, known as stress hyperthermia [24], a phenomenon that is present in other species such as laboratory rats [50,51] and sheep [46]. In canines with separation anxiety, IRT applied in the same thermal window has detected an increase in ocular temperature when the owner is absent; however, an increase was also registered when the animal was in contact with the owner. These results show that, although ocular temperature responds to autonomic activity, it can have positive and negative valence [52].

This situation was discussed in a complementary study by Travain et al. [6], which analyzed the emotional responses of 19 dogs to potentially pleasant events (the presence of food in the form of candies or kibble) by evaluating their physiological (cardiac frequency) and cardiac responses (heart rate variability), and behavior, in addition to using IRT. Those authors observed that the temperature of the lacrimal caruncle and the cardiac frequency increased during positive stimulation but with no modification of heart rate variability. They also reported behaviors associated with excitation that occurred in the presence of the candies, manifested by greater tail movement. 

The scientific evidence shows that the lacrimal caruncle’s temperature has significantly increased due to the activity of the ANS associated with potentially stressful events, being a non-invasive indicator of this state or negative emotions such as anxiety or fear in companion animals [41,53]. Likewise, Travain et al. [54] mention that IRT on the ocular surface could also help study the temperament of animals due to the lateralization of blood circulation that results in different thermal patterns, depending on the reaction of the individual, the nature of the stressor, and the previous animal experiences.

**Figure 3:**
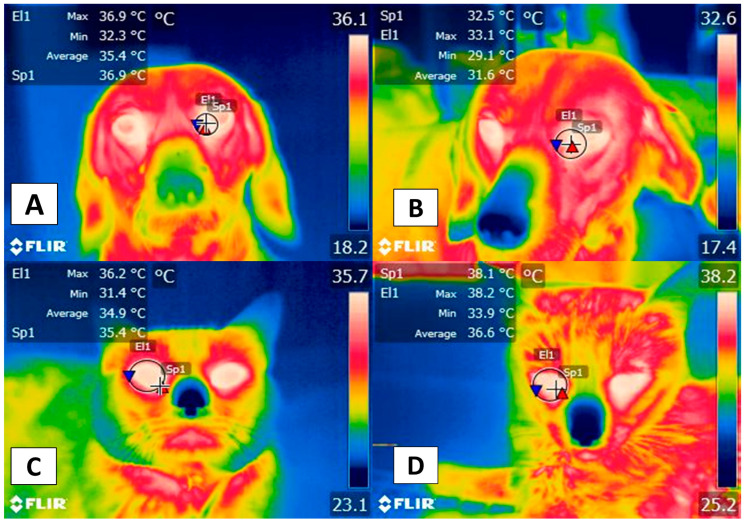
Facial thermal windows in the dog and cat: (**A**) Lacrimal caruncle. The lacrimal caruncle (El1) is shown in the eye region of a 4-year-old dog prior to surgery. This area runs from the medial commissure of the eyelid to the mid-region of the eye. Additionally shown is the temperature of a dog at rest. The maximum temperature was 36.9 °C (red triangle), the minimum was 32.3 °C (blue triangle). (**B**) Lacrimal caruncle (El1). This image shows modifications in the same dog after 2 h of concluding the surgical procedure with a decrease of 3.8 °C from the maximum (33.1 °C, red triangle) and 3.2 °C from the minimum (29.1 °C, blue triangle) generated by the perception of pain during the surgical procedure that activated the ANS, causing a reaction that consisted of peripheral vasoconstriction with a decrease in surface circulation that reduced the irradiated temperature. This would explain the thermal reaction observed. (**C**) Ocular region. Image showing the ocular thermal window (El1) of a 4-year-old feline wounded by the attack of a conspecific. This thermal window covers the entire interior zone of the eyeball. The maximum ocular temperature recorded was 37.3 °C (red triangle), the minimum was 33.9 °C (blue triangle) while the animal was comfortable or at rest. (**D**) There is an increase of 0.9 °C in the maximum temperature (38.2 °C, red triangle) due to peripheral vasodilation. This vasomotor response is a consequence of proinflammatory mediators (e.g., histamine) released after the injury. Sp1: default focal point of the software. Thermal images obtained using a FLIR thermal camera.

There are no studies of canines similar to Stewart et al.’s work [47] with 30, 4-month-old bovines after castration, which established the presence of a cardiovascular response associated with pain due to activation of the SNS and found that the increase in the surface temperature of the lacrimal caruncle was related to higher blood pressure and cardiac frequency. Those authors also observed that this occurred synchronically with an increase in blood catecholamine levels, indicating that the thermal response of the lacrimal caruncle recorded by IRT is closely related to the activity of the ANS. The above mentioned is similar to observations of the authors in dogs (Figure 3). These results suggest that this technique and region could indirectly register autonomic activity in animals, unlike observations in humans [9]. 

Zanghi [22] reported that the temperature of both the eye and auricular region showed a direct relation (r^2^ = 0.67 eye, r = 0.61 ear) to rectal temperature in 32 dogs during exercise. They observed that rectal, eye, and auricular region temperatures decreased significantly as the exercise duration increased. These events differ to what Elias et al. [12] recently found when evaluating thermal stress and vascular responses before and after exercise in 465 greyhound dogs by recording average eye temperatures. They discovered that the eye temperature increased after exercise and, also, that the temperature of the right eye was more sensitive to changes but suggested the need to consider environmental and biological factors when detecting stress during exercise. 

These findings prove that circulation in specific structures—especially the eye—responds more to ANS activity than the auricular region. Thus, it presents an effective technique for demonstrating degrees of stress in animals.

Regarding the relation of IRT to pain evaluation scales, the results are discrepant. For example, Lush and Ijichi [5] evaluated the behavioral and physiological responses of 20 dogs after orchiectomy. They affirmed that the temperature of the lacrimal caruncle could not be associated with pain scores (r = −0.107), though they did observe a temperature increase in that region. A separate study conducted on 30 bitches subjected to ovariohysterectomy under three epidural analgesia protocols reported that the temperature of the lacrimal caruncle did not show significant differences between groups. This effect was attributed to the analgesia provided, an element that was corroborated with the score obtained in two postoperative pain scales (the Dynamic Interactive Visual Analogue Scale and the University of Melbourne pain scale) [55]. While this led to the observation that the lacrimal caruncle presents vascular and thermal responses that accord with ANS activity, it raises questions about IRT’s sensitivity and specificity when used in this region. It is important to note that some authors sustain that optimizing evaluations of thermal responses during painful events requires performing IRT in association with facial expressions. This approach helps determine the degree of pain during surgical processes or periods of intensive care. As expression intensity is accentuated, it can be correlated with thermal responses at a sensitivity of 75% [56,57].

Evidence for eye temperature relevance in cats is clear, at least according to Foster and Ijichi’s [41] report on an evaluation of 34 cats in stress situations under distinct breeding conditions in which IRT was applied to the central region of the eye. Those authors observed that temperature correlated significantly with acceptable scores for the profile of feline temperament (r^2^ = −0.37, *p* = 0.028) and established that eye temperatures were higher in older cats and those housed individually than those kept in groups. 

Another field of evaluation of thermographic responses in cats involves stress induced by separation from the owner. Observations showed that the auricular region temperature decreased during separation but registered an increase as soon as the owner returned to contact with the patient [42]. The thermal window of the auricular pavilion (pinna) has also been used in companion animals to evaluate responses to stress in these species. Reimer et al. [42] examined the ear temperature (in both ears) in six dogs subjected to a 2-minute-social isolation test from the owner. The temperature in both ears decreased by 0.2 to 0.6 °C during separation and increased in the owner’s presence (between 0.4 and 0.7 °C). In contrast, the opposite effect has been observed in cats. In a study conducted on 41 domestic felines from one to eight years old, the animals were divided into two groups according to serum cortisol levels (high: 7 mcg/dL, and low: 3 mcg/dL) and were exposed to transport and an unknown environment to quantify the ear temperature. After the stimulus, the temperature (0.19 °C, *p* < 0.04), glucose, and cortisol increased significantly; however, a correlation between both factors could not be established. The authors also reported that the right ear but not the left ear temperature is related to stress-induced cortisolemia [58]. 

These results lead to questioning the usefulness of the thermal windows and IRT in general. As Polgár et al. [59] mentioned, thermography can be influenced by a wide variety of factors that could alter the accuracy to assess the valence of the emotional state of animals. IRT indicates a level of arousal and autonomic activity but does not assure that increases or decreases in temperature are attributed to a negative or positive stimulus. In contrast, in dogs, tympanic membrane temperature has been suggested as a suitable method to monitor temperature, as it is closely related to the standard method of rectal measurement, with a mean difference of 0.39 °C between both [60]. 

Sousa et al. [61] measured the ear temperature to estimate the standard rectal method in cats. In this study, 29 healthy cats were evaluated for two weeks, observing that ear temperature maintained a direct correlation with rectal temperature (r^2^ = 0.96). Likewise, in 32 dogs, it has been observed that the ocular and auricular temperature maintained a direct correlation with the rectal temperature (r^2^ = 0.67 eye, r^2^ = 0.61 ear), considering the auricular region as an area that reflects accurately thermal rectal values [22]. In contrast, in a study conducted on 19 domestic cats, the ear and rectal temperatures had a weak correlation (r^2^ = 0.62) with mean differences of 0.07 °C and limits of 1.43 °C [62]. These results are similar to those obtained by Nutt et al. [63], who evaluated the temperature in three anatomical regions (pinna, perineum, and gingiva) of 188 domestic shelter cats. A poor agreement was observed between ear IRT and rectal temperature using Bland–Altman analysis; furthermore, the mean values ranged from 0.7 to 1.3 °C. The researchers suggest that this tool does not offer reliable values to support its clinical use in the aforementioned regions. The main limitation reported in the ear is the presence of hair or secretions in the external auditory canal that can alter the values readings and interpretations [42,62,64].

The nasal plane has been used to determine the degree of comfort of canines undergoing radiotherapy for intranasal tumors. In a clinical case report of a shikoku inu dog (a breed native to Japan), nasal IRT showed an increase of up to 4.1 °C after radiotherapy, reaching a maximum temperature of 42.3 °C. In humans, the increase in skin temperature after such treatments is associated with stress and pain. However, in the shikoku inu dog used in the study by Saeki et al. [65], the lack of behaviors associated with pain or stress in the patient, and the late response of the increase in temperature (120–140 min after radiotherapy) did not establish a relationship between hyperthermia and pain. Despite these findings, other authors recommend evaluating the entire facial region, or the face itself, since observations in human medicine—according to recent reports based on assessments spanning the ears, nose, lips, and cheeks—could be associated with the degree of comfort that an individual feels at a precision as high as 85% [43,66]. Considering the entire facial region or specific thermal windows could represent a complimentary assessment through IRT. However, some environmental and technical factors must be considered before and during the application of IRT in animals. 

Although the evidence and the use of IRT make it a useful complementary tool for clinical practice in veterinary medicine, in both humans and animals, environmental, individual, and technical aspects are reported as factors that can influence the values obtained by the IRT [67]. Solar radiation, humidity, and wind speed in animals can affect their reading. For example, direct solar radiation can increase the temperature of the different thermal windows by up to 0.5 °C, similar to what was reported by Loughmiller et al. [68], who observed in pigs that environmental temperature had a significant linear relationship with body surface temperature (slope value = 0.40 °C). 

This environmental temperature influence has shown in equines a relationship with the IRT measured in the carpus region (r^2^ = 0.88) [69], so that the climate conditions are a factor that, preferably, must be controlled to avoid variations. This has been applied in dogs by controlling the room temperature at 21 °C to unify the characteristics and obtain a reliable IRT reading [11].

The presence of wind is another factor that, contrary to solar radiation, can cause the ocular surface temperature to drop by 0.7 °C [70]. Therefore, these environmental factors can influence the IRT reading and must be considered or controlled by the evaluators for a correct results interpretation. In addition, biological elements such as the different species, the presence of hair, fur color, and the presence of fluids may also need to be evaluated, together with laterality.

The technical aspects are another variable that must be considered when using thermographic cameras. In humans, it is reported that technical factors such as the type of camera and the degree of resolution can affect the temperature measurement. According to some authors, the resolution of 320 × 240 pixels is recommended since it allows the detection of minimal changes in surface temperature, a process that is difficult with low image resolutions [2,15,71]. This factor is also closely related to the camera lens type since some lenses are coated with diamond or germanium films that allow infrared radiation to be captured, while other types of cameras with a compound lens system process the infrared radiation in an electronic circuit [2,72]. 

Therefore, it is important not only to establish the thermal window, recognizing that the eye region—the lacrimal caruncle or ocular area—is especially efficacious for determining the degree of thermal equilibrium under stress when compared to the ear, but also considering the type of camera and external factors that can alter IRT readings in companion animals.

## 4. Appendicular Windows

**Figure 4:**
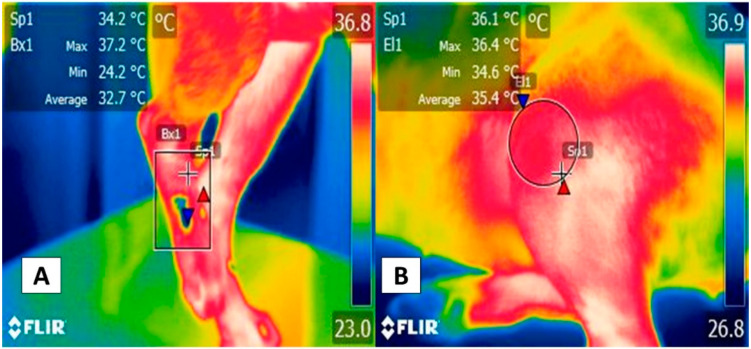
Modified thermal windows in forelimbs and hindlimbs for complementary diagnoses of inflammatory pathologies: (**A**) Complementary diagnosis of a metacarpal fracture. The rectangular figure (Bx1) shows a significant temperature increase in the metacarpal region of the right pelvic limb with a maximum of 37.2 °C (red triangle) and a minimum of 24.2 °C (blue triangle). This was associated with a fracture of the metacarpal bones in a 4-year-old male boxer dog. It is important to note the minimum temperature reduction, which could be related to a lack of local circulation produced by the destruction of surface blood vessels. In contrast, the maximum temperature is associated with the inflammation of peripheral tissues. (**B**) Complementary diagnosis of articular pathologies. The circular figure (El1) shows the significant increase in temperature with a maximum of 36.4 °C (red triangle) and a minimum of 34.6 °C (blue triangle) in the tibia-femoral-patellar joint of a male Pitbull dog with a cranial cruciate ligament rupture, manifesting a <3 C° limp or surrender, in the left pelvic limb. Both cases show that IRT can provide a complementary, non-invasive method that aids in initial approaches to patients that have suffered trauma. Sp1: default focal point of the software. Thermal images obtained using a FLIR thermal camera.

In addition to the facial thermal windows just discussed, the thoracic (*membri thoracici*) and pelvic limbs (*membri pelvini*) have been proposed for evaluating specific pathologies, such as inflammatory problems (Figure 4). The hip (*regio articulationis coxae*), shoulder (*regio articulationis humeri*), knees (*regio genus cranialis*), and elbows (*regio olecrani*) are regions susceptible to changes in the emitted radiation. In the case of the thoracic limb, this region is supplied by the axillary artery (*arteria axillaris*) emerging from the first rib and continuing as the deep brachial artery (*profunda brachii*); in the forearm as the deep ante-brachial artery (*profunda antebrachii*), and is divided into various branches at the distal level: in the palmar carpus (*ramus carpeus palmaris*) and the palmar (*ramus palmaris*) [45]. 

**Figure 5:**
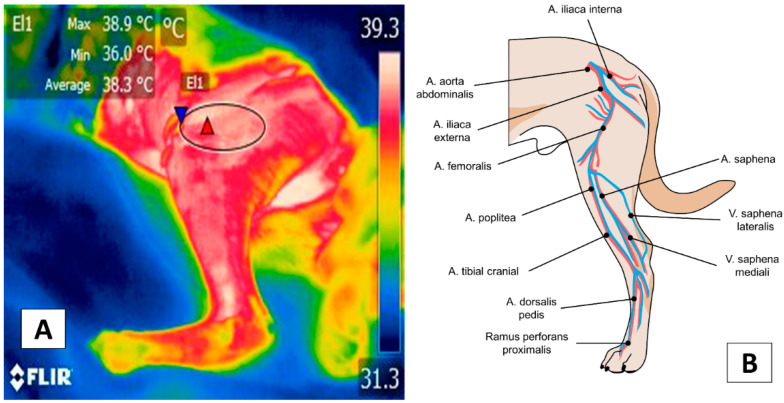
Appendicular window: (**A**) Femoral thermal window (El1). The tracing of this window is through an ellipse of approximately 3 cm located in the cranial region where the quadriceps femoris muscles (*quadriceps femoris*) lie and considering the cranial region of the tensor fasciae latae (*tensor fasciae latae*). (**B**) Circulation at the femoral level. The femoral thermal window obtains its circulation from the femoral artery (*arteria femoralis*) and its branch with the lateral circumflex artery (*circumflexa femoris lateralis*) that nourishes the previously described muscles that confer the importance of this window for the evaluation of the temperature. Thermal images obtained using a FLIR thermal camera.

Figure 5 shows the vascularization of the pelvic limb. This is derived from the external iliac artery, derived from the femoral artery (*femoralis*) that, at the medial level of the femoral region (*regio femoralis*), continues as the saphenous artery (*saphena*) and divides into the caudal femoral artery distal (*caudalis femoris distalis*) and in the caudal tibial (*tibialis cranialis*) and cranial (*tibialis caudalis*) artery [45].

The high density of blood vessels in the fore and hindlimbs permits visualization of the heat exchange through IRT, particularly during exercise. Rizzo et al. [73] evaluated the surface and body temperature of ten Jack Russel Terriers during 15 min walks, 10 min jogs, and 10 min gallops. After each event, the superficial temperature of the neck, flanks, ribs, back, inner thigh, and eye was collected. The results showed that the superficial temperature in the inner face of the thigh and the eye had a significant increase (*p* < 0.0001), together with increases in rectal temperature, hemoglobin levels, hematocrit, and red blood cells counts during the jogging phase (*p* < 0.05), due to the increase in blood flow in response to muscle activity. A similar event has been reported in the gastrocnemius, *biceps femoris*, and *gracilis* muscles in healthy dogs walking for 11 min. When applying a separate analysis of each muscle region, the *biceps femoris* and *gracilis* showed an increase in temperature after walking, unlike the gastrocnemius muscle; furthermore, a significant difference was detected between the temperatures of the right and left extremity [74].

Sturion [75] also applied the study of muscle groups and joints, together with cortisol levels, to determine the levels of stress and pain present in animals after performing the exercise. The author found a positive correlation between elevations in IRT, serum cortisol levels, and tympanic temperature. Therefore, detecting alterations in the muscular blood flow of broader regions such as the thoracic and pelvic limbs offers information about the muscular activity.

One study that postulates the use of appendicular thermal windows was conducted by Sung et al. [20], who evaluated 40 dogs using thermal windows in areas of the shoulder, elbow, paw, hip, knee, and hock. They were able to identify surface temperature differences between the extremities affected by bone neoplasms and reported a statistically significant difference in the average temperature between the regions of interest in their study groups (0.53 ± 0.14 °C) with a success rate of 75%–100% for identifying bone injuries.

In contrast, specific thermal windows such as those of the joints have not produced conclusive findings [15]. This was reported by Alves et al. [11], who collected 900 images of 50 work dogs, which considered the region from the last lumbar vertebra to the first coccygeal vertebra in dorsal position, while in the lateral direction, they focused on the greater trochanter region in the center. Those researchers determined that the temperature of the trochanter region was significantly higher (28.5 ± 2.8 °C) than the dorsal region (25.3 ± 9.1 °C) and mentioned a low correlation between these two regions (r^2^ = 0.10, *p* = 0.03). These findings demonstrate that a joint such as the hip offers a thermal window that is more sensitive for diagnosing joint diseases in dogs. 

Findings for cats are similar since correlation analyses of thermographic results of the region of affected tissue with the histological subtype and tumor degree in 11 cases of feline sarcoma and 31 patients with tumors in the skin or soft tissues showed that mean temperature was significantly higher in malignant tumors than benign ones (35.4 ± 01.8 °C vs. 34.4 ± 1.7 °C), and those temperatures above 34.7 °C could be associated with malignancy. That study reported a sensitivity of 76% and a specificity of 80% (*p* = 0.01) [76]. Studies performed to date in this species and dogs agree that IRT can be a sensitive, efficacious tool for clinically differentiating malignant from benign tumors and could aid in arriving at timely prognoses in oncological patients. 

**Figure 6:**
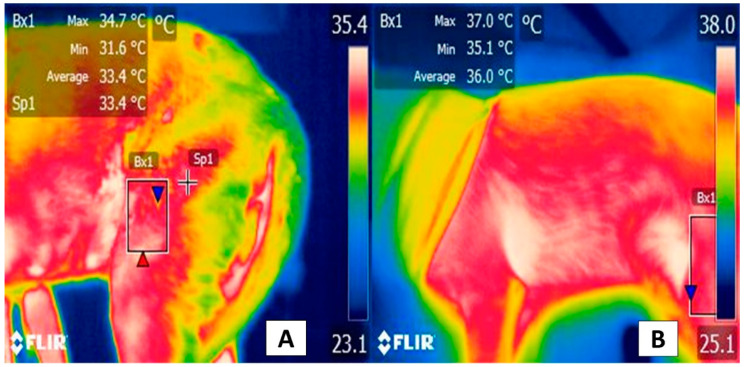
Thermal response of the femoral biceps muscle during moderate exercise. The thermal window of the femoral biceps muscle has been proposed to determine dogs’ thermal state during exercise. This muscle spans the lateral face of the cranial zone of the femoral region, from the middle third of the femur to the proximal level of the distal epiphysis, 3 mm from the tibia-femoral-patellar joint. This zone extends to the head surface of the femoral biceps that, finally, has a tendinous insertion into the tibial tuberosity, represented by the rectangle (Bx1). (**A**) Thermal response of the femoral biceps muscle prior to exercise. This image shows the thermal response of the femoral biceps muscle (Bx1) with maximum and minimum temperatures of 34.7 °C (red triangle) and 31.6 °C (blue triangle) in a 3-year-old, non-breed female at rest. (**B**) Thermal response of the femoral biceps muscle after moderate exercise. The image shows the thermographic response after 15 min of moderate exercise during a clinical assessment of the dog’s march. The significant increases of 2.3 °C (red triangle) and 3.5 °C (blue triangle) in the maximum and minimum temperatures stand out. It is suggested that this response is due to the increase in dogs’ central temperature during physical activity, and hence the femoral biceps muscle receives significant vascularization from the femoral artery. Sp1: default focal point of the software. Thermal images obtained using a FLIR thermal camera.

A similar response has been reported in evaluations of more specific appendicular regions, such as the gastrocnemius, *biceps femoris*, and *gracilis* muscles. For example, in healthy dogs during an 11 min walk, the analysis of individual muscles showed that the femoral biceps and *gracilis* presented temperature increases after the walk that were not evident in the gastrocnemius muscle. In addition, those authors observed a significant difference between the right and left limbs [74]. Thus, isolated muscle structures could provide an additional, more exact, window for evaluating thermal and vascular responses to exercise (Figure 6).

However, controversy has emerged because some studies report the presence of a significant influence related to the type of fur (short vs. long) that could alter IRT evaluations [77,78]. According to Nomura et al. [79], IRT was used as a complementary method in evaluating the condition of the knees of 30 dogs using the lateral, cranial, and medial faces of the joint. Those authors found no differences in the dogs’ facial expressions during their assessment of this anatomical region but did observe that the temperature was 3 °C higher in the dogs with short hair than those with long hair. 

This observation was reaffirmed by Kwon et al. [13] in their evaluation of the influence of the characteristics of animals’ hair on the body surface, using 50 dogs acclimated to solar light for 15 min. They later obtained images of the lateral face of the femoral region and determined that the animals with a long, double coat of hair had lower temperatures (28.14 ± 0.31 °C and 28.25 ± 0.23 °C) than those that had short hair (31.77 ± 0.19 °C). Those authors concluded that this feature must be considered when evaluating appendicular extremities in this species using IRT. Something similar has been reported in horses, in which a greater density of fur was associated with a lower shoulder temperature (*p* < 0.001) [80].

Despite these discordant results, it has been suggested that IRT could aid in detecting temperature decreases instead of increases. This proposal is related to hemodynamic or ischemic alterations, where the literature includes reports of a negative correlation between blood pressure and average and surface temperatures based on readings from the elbow and carpal region in a porcine model [81]. Those findings indicated the need to evaluate the diagnostic precision of IRT in cats with acute paralysis to differentiate between aortic thromboembolism and non-ischemic conditions. That study reported that the mean, maximum, and minimum temperatures were lower in the right pelvic limb than on the left side. Moreover, the authors successfully identified that a temperature reduction of less than 2.4 °C in the pelvic limbs was the point for differentiating ischemia compared to controls. That work registered a sensitivity of 90% and a specificity of 100%, with positive and negative predictive values of 100% and 86%, respectively [33].

In cats, the findings with IRT are similar to those in dogs. Quantifying the surface temperature at the limbs’ levels can help detect lesions, as seen in the study by Vainionpää et al. [82], who evaluated 103 cats diagnosed with joint-level injuries. These animals underwent a physical examination, IRT valorization, and an owner questionnaire. The frequency of injuries, examined through the IRT, presented a moderate agreement of 0.48 according to a weighted kappa analysis with a confidence interval of 95%, while a low agreement was observed between owner detection of pain and thermographic images. Particularly in felines, implementing these non-invasive technologies is postulated as a valuable alternative for stress-free management during daily clinical practice [54].

Finally, the literature has suggested that these windows can help determine the degree of thermal comfort in neonatal animals. According to Reyes-Sotelo et al. [7], the low or null capacity to thermoregulate, the evaporation of the amniotic fluid, and the contact with cold surfaces are causal factors of hypothermia during this stage. In neonates, the femoral and the shoulder region are postulated as potential thermal windows [83], as indicated in humans [84].

Because of the nature of this corpus of evidence, the clinical use of appendicular thermal windows is not yet widely disseminated, so its effectiveness compared to the facial region is not clear because of characteristics such as hair type and environmental factors that reduce the reliability of these windows. Likewise, laterality probably plays a more significant role in this region since evidence indicates that the right region is more sensitive than the left. This topic must be considered in future studies.

## 5. Body Windows

Unlike the facial or appendicular thermal windows, thermal body windows have not been described with great exactitude, as in the case of ruminants [35,38] or pigs [85], species in which the thorax (*regio pectoralis lateralis*) or the abdomen (*regio abdominis lateralis*) are proposed as possible thermal windows to estimate the degree of comfort when exposed to extreme environmental conditions [86]. However, certain modified regions have been explored to evaluate surface lesions, as in the case of descriptions of tumors [20]. In this regard, Pavelski et al. [21] reported that the surface temperature of the mammary gland—inguinal or abdominal—was higher when affected by tumors than healthy glands. This finding suggests that IRT could function as a method for the early detection and localization of malignancy in mammary gland tumors. However, these observations await corroboration in veterinary practice, especially in clinical medicine with dogs and cats. When a malignant tumor develops, the presence of prostaglandins, serotonin, histamine, and tumor necrosis factor causes peripheral vasodilation, an event that is not observable in benign tumors [87,88]. Even though IRT is an innovative method that makes it possible to detect early changes, the lack of sensitivity and specificity studies when applied to specific body regions has reduced trust in the instrument. For this reason, one option proposed consists of complementing IRT with other clinical tools. IRT is especially sensitive to changes in the surface dermal microcirculation that occurs during skin and inflammatory diseases, and neoplasms, among other conditions [89].

**Figure 7:**
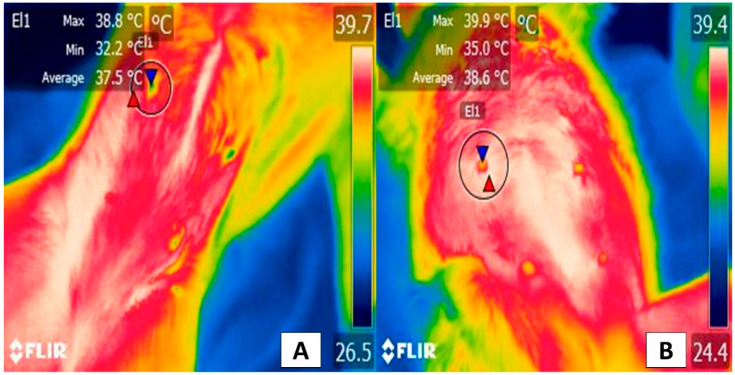
The thermal window of the mammary gland: (**A**) Healthy gland. The window of the mammary gland (El1) is shown in a 4-year-old Doberman Pinscher female dog. A maximum temperature of 38.8 °C (red triangle) and a minimum of 32.2 °C (blue triangle) can be seen. (**B**) Gland with the presence of tumors. An 8-year-old female dog of the Maltese breed with 0.5 to 1 cm diameter tumors in the left abdominal gland (El1). In this region, the temperature is 1.1 °C higher in the maximum values (red triangle), while the minimum is 2.8 °C higher (blue triangle), compared to the temperature shown in a healthy gland. The increase in temperature at the local level is due to cancer cells releasing pro-inflammatory substances such as serotonin, histamine, prostaglandin F2 α, and tumor necrosis factor α promoting vasodilation of superficial blood capillaries, causing an increase in the radiation emitted. Thermal images obtained using a FLIR thermal camera.

The clinical application of IRT to detect malignant tumors was studied in 11 cats with feline sarcoma and 31 patients with skin or soft tissue tumors. Mean temperature was significantly higher in malignant tumors compared to benign ones (35.4 ± 01.8 °C vs. 34.4 ± 1.7 °C), and when the temperature was higher than 34.7 °C, this finding was associated with the malignancy of the tumors, with a sensitivity of 76% and a specificity of 80% (*p* = 0.01) [76]. In this way, both in dogs and cats, the studies agree that IRT represents a sensitive and effective tool for the clinical differentiation between malignant and benign tumors, contributing to estimating a timely prognosis in oncological patients (Figure 7).

Other studies have established the use of specific thermal windows, such as the ventral aspect of the neck. For example, Waddel et al. [90] evaluated 17 cats’ hypothyroidism and 12 healthy cats by recording thermographic changes before and after cutting the animals’ hair. In addition, those patients were administered radioiodine to evaluate responses at 1- and 3-month post-treatment. Authors reported an IRT precision ranging from 80.5% to 87.5% for detecting the animals with hypothyroidism before and after the hair over the ventral aspect of the neck was clipped. It might have been possible to corroborate their findings by measuring serum thyroxine levels. 

These results are similar to those observed in 15 Anatolia Shepherd bitches in which thermal images were taken of the perianal and perivulvar regions to determine whether IRT could detect estrus in this species. That study determined that the surface temperature of these regions presented a non-significant increase related to serum progesterone levels and percentages of keratinized surface cells. Despite these results, the authors state that IRT could be a complementary tool for determining estrus in female dogs [91]. In addition, the authors suggest that further studies are required to determine whether IRT, as in the case of cattle [92] and pigs [93], can serve as a complementary tool to assess the reproductive status of small animals.

The inguinal region and the gum are other body regions studied with IRT. These have been shown to correlate moderately with the rectal temperature of 204 canines (*p* < 0.001 and *p* < 0.001, respectively), and, in particular, the gum temperature showed differences of 1 °C when compared to the rectal temperature, obtaining a sensitivity of 90% and a specificity of 78.6% to detect hyperthermia [94]. In contrast, another potential use of IRT in companion animals could be for early detection of hypothermia in areas where postural ulcers are usually present due to paralysis of the pelvic limb or severe spinal cord damage, as in human medicine [95].

This broad panorama leads to the inference that the results of these isolated studies of thermal body windows are inconclusive. Then, tasks for future research must include establishing the degree of reliability of IRT testing and determining the exact windows where specific conditions can be detected. Recent reviews suggest that windows such as the thoracic region, complemented by thoracic and pelvic limbs analyses, could be an effective method for evaluating thermal states in neonate canines, similar to findings in human medicine [7,84]. 

Therefore, body regions may have additional considerations for utilizing the IRT technique (e.g., type of pathology) over and above the factors mentioned about the other windows since the evidence currently available is not conclusive, even though various applications have been reported. The studies analyzed herein on body regions suggest that areas such as the neck, perivulvar, and perianal zones could be helpful to thermal windows, but it is necessary to establish their respective degrees of reliability.

## 6. Conclusions

The current importance of IRT demonstrates its usefulness for the early, non-invasive diagnosis of diverse inflammatory conditions, while results from some studies indicate that it could be an important method for detecting and differentiating malignant vs. benign neoplasms. Nonetheless, studies of regions such as the lacrimal caruncle, eye, or auricular region in dogs and cats have shown that these zones are especially sensitive to the activity of the ANS associated with stress factors. However, today’s evidence demonstrates that the ocular region is a validated thermal window for cats with high sensitivity and specificity. For this reason, it is necessary to establish whether this is true for dogs and other potentially useful thermal windows. In addition, it is urgent to analyze biological and environmental factors concerning thermographic evaluations of different anatomical regions.

Regarding the appendicular and body thermal windows, the factors mentioned more strongly influence temperature dispersion, though one advantage is that they supply more information on the conditions of the thermal state of animals during illness. It is important to mention that using isolated regions such as the femoral biceps and *gracilis* muscles can provide more precise information on thermal states after exercise or on local inflammatory conditions in dogs. This technique has shown high sensitivity even in low circulation conditions in felines.

Finally, the body and appendicular regions discussed show the greatest contrast and discrepancies in their potential clinical use compared to facial windows. Therefore, additional research is required to establish the sensitivity and specificity of these areas for a possible future validation of the use of IRT in dogs and cats.
